# Mucosal ulcers, extremity nodules, and fevers

**DOI:** 10.1016/j.jdcr.2024.10.017

**Published:** 2024-11-07

**Authors:** Hannah G. Steffke, Michael S. Chang, Ashley Ward, Christopher Iriarte

**Affiliations:** a Harvard Medical School, Boston, Massachusetts; b Department of Dermatology, Beth Israel Deaconess Medical Center, Boston, Massachusetts; c Department of Pathology, Beth Israel Deaconess Medical Center, Boston, Massachusetts

**Keywords:** autoinflammatory vasculitis, Behçet’s disease, Epstein-Barr virus, erythema nodosum

## History

**Fig 1:**
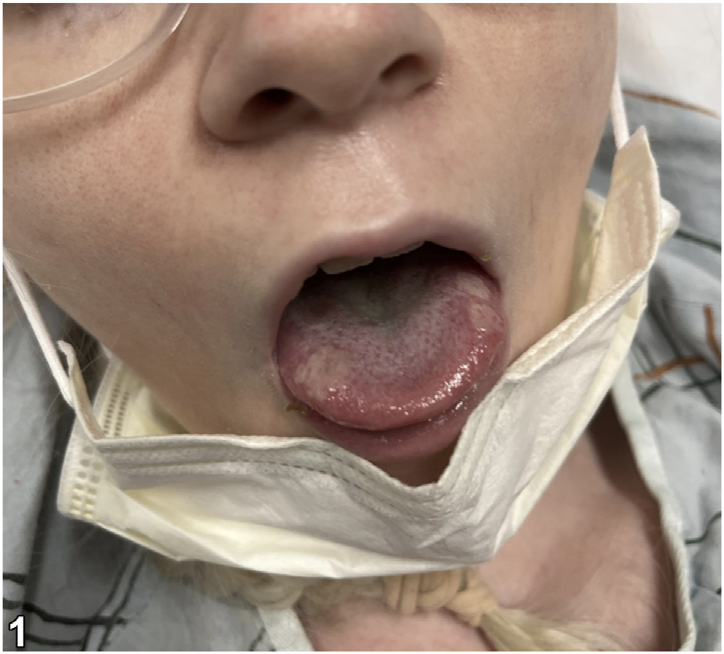

**Fig 2:**
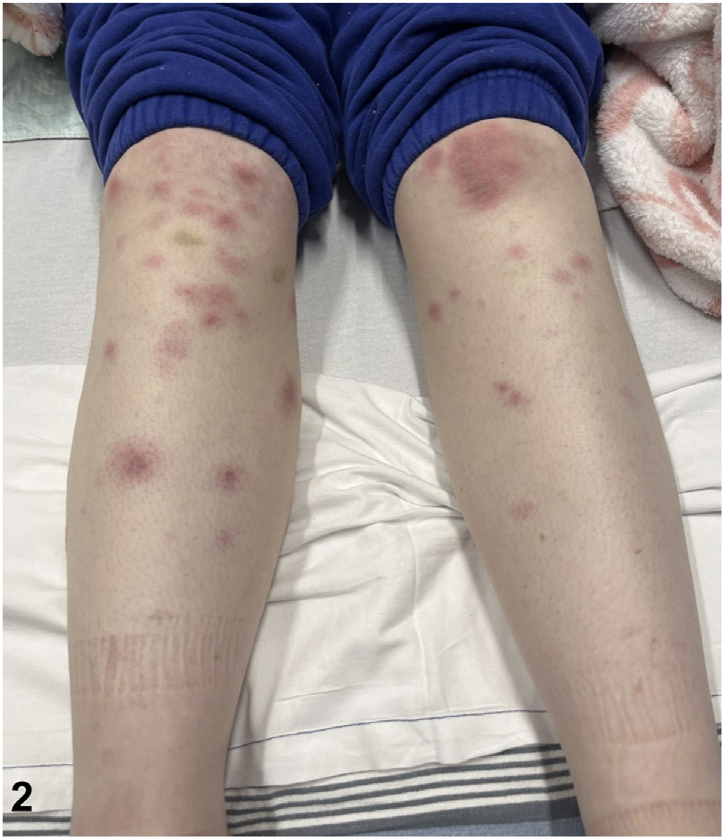

**Fig 3:**
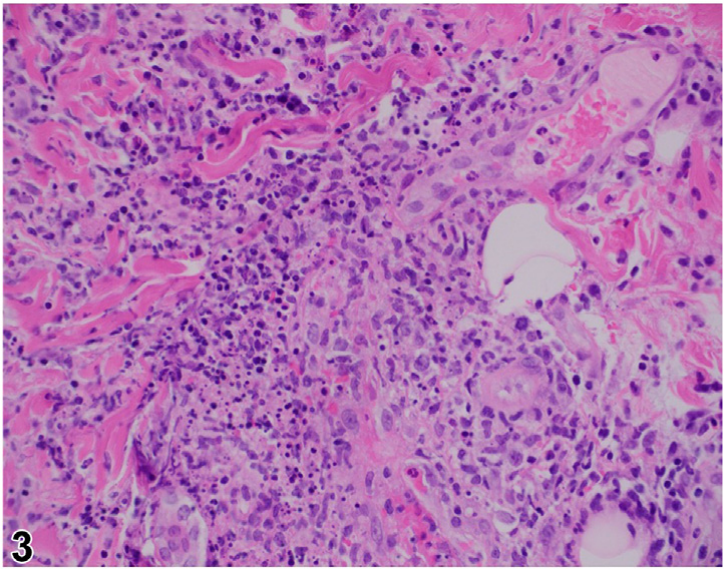

A 27-year-old female presented initially with several days of profound fatigue, cervical lymphadenopathy, and fevers. She was prescribed a course of amoxicillin-clavulanate before re-presenting 1 week later with fever, headaches, photophobia, arthralgias, diarrhea, dysuria, and painful skin lesions. Examination revealed oral and labial ulcers (Fig 1), erythematous nodules on the extremities (Fig 2), conjunctival hyperemia, papilledema, and vulvar erythema. Laboratory studies were notable for neutrophilia, anemia, elevated C-reactive protein, and positive Monospot and Epstein-Barr virus (EBV) viral capsid antigen IgG. Infectious and autoimmune workup was otherwise unremarkable. Histologic findings from biopsy of a subcutaneous nodule are shown (Fig 3).

**Question 1: What is the most likely diagnosis?**

A. Sweet syndrome
B. Behçet’s disease
C. Primary EBV infection
D. Erythema multiforme
E. Primary Herpes Simplex Virus (HSV) infection

**Answers:**

A. Sweet syndrome – Incorrect. Sweet syndrome presents with neutrophilia, fevers, and typically edematous plaques or vesiculobullous lesions. Histology would show a dense neutrophilic infiltrate and papillary dermal edema without evidence of leukocytoclastic vasculitis.
B. Behçet’s disease – Correct. The oral and genital ulcers, neutrophilic vasculitis on histology of erythema nodosum-like skin lesions, as well as ocular, neurologic, gastrointestinal, and joint symptoms with an otherwise unremarkable infectious and inflammatory workup led to a diagnosis of Beçhet’s disease.
C. Primary EBV infection – Incorrect. EBV infection may have been both an inciting trigger for Behçet’s disease and the cause of her initial presentation consistent with infectious mononucleosis. Associations between EBV infection and Behçet’s disease have been well demonstrated.^1^ While EBV itself can have dermatologic manifestations, EBV alone was improbable, particularly given evidence of neutrophilic vasculitis on biopsy and subsequent EBV viral load and IgM testing were negative.
D. Erythema multiforme – Incorrect. The rash associated with erythema multiforme typically consists of targetoid plaques on the distal extremities. Skin biopsy findings here are also not compatible with erythema multiforme, which would show a vacuolar interface dermatitis with a lymphocytic infiltrate, rather than a neutrophilic vasculitis as pictured.
E. Primary HSV infection – Incorrect. Erythema-nodosum like skin lesions would be highly unusual for primary HSV infection. HSV serologies and PCR were obtained and negative.

**Question 2: Which of the following is most often associated with Behçet’s disease?**

A. HSV infection
B. Streptococcal infection
C. Human leukocyte antigen (HLA)-B27
D. HLA-B51
E. Anti-nuclear antibodies

**Answers:**

A. HSV infection – Incorrect. Behçet’s disease has been hypothesized to be triggered by a preceding infection. The most commonly implicated infections include HSV-1 and streptococcus, but multiple infections, including EBV, suspected in this case, have been previously associated.^1^^,^^2^ Infections are however not a stronger risk factor than HLA-B51.
B. Streptococcal infection – Incorrect. As above, infections are not the strongest risk factor for the development of Behçet’s disease.
C. HLA-B27 – Incorrect. HLA-B27 is independently associated with an increased risk of Behçet’s disease, however is it not the strongest associated haplotype associated with this condition.^3^ HLA-B27 is more strongly associated with increased risk other autoinflammatory conditions, including ankylosing spondylitis, inflammatory bowel disease, and psoriasis.^3^
D. HLA-B51 – Correct. HLA-B51 is the most well-established predisposing genetic factor associated with Behçet’s disease. It is, however, not necessary, specific, or sensitive for diagnosis, especially in patients that are not of Northern European descent.^3^
E. Anti-nuclear antibodies – Incorrect. These antibodies are not associated with risk of developing Behçet’s disease and are implicated in a varie ty of connective tissue diseases such as lupus, scleroderma, and dermatomyositis.^4^

**Question 3: What feature of this patient’s presentation is associated with neurologic involvement of Behçet’s disease?**

A. First presentation
B. Female sex
C. EBV trigger
D. Normal lumbar puncture
E. HLA-B51 status

**Answers:**

A. First presentation – Incorrect. For patients with Behçet’s disease with neurologic involvement, the median time elapsed between initial presentation and neurologic involvement is 6 years.^5^
B. Female sex – Incorrect. The prevalence of neuro-Behçet’s disease is generally increased among males, not females, although some studies show a female predominance in Portugal and northern Spain.^6^
C. EBV trigger – Correct. EBV viral capsid antigen IgG titers, which were present in this patient, have been associated with eventual neurologic involvement of Behçet’s.^2^ Neurologic sequelae of Behçet’s disease vary but include both direct parenchymal and nonparenchymal involvement (eg cerebral venous sinus thrombosis, cerebrovascular attack, pseudotumor cerebri). Our patient had neurologic involvement as manifested by her headaches and papilledema.
D. Normal lumbar puncture – Incorrect. For patients with parenchymal neurological disease, most will have pleocytosis or elevated protein levels in the cerebrospinal fluid, while those with nonparenchymal involvement tend to have elevated opening pressures.^5^
E. HLA-B51 status – Incorrect. HLA-B51 status has not generally been shown to correlate with presence of neurologic sequelae of Behçet’s disease.^6^

## Conflicts of interest

None disclosed.
